# Assessment of the Multi-Objective Reservoir Operation for Maintaining the Turbidity Maximum Zone in the Yangtze River Estuary

**DOI:** 10.3390/ijerph15102118

**Published:** 2018-09-26

**Authors:** Yang Yu, Peifang Wang, Chao Wang, Xun Wang, Bin Hu

**Affiliations:** 1 Key Laboratory of Integrated Regulation and Resource Development on Shallow Lakes, Ministry of Education, Hohai University, Nanjing 210098, China; yuyang_hhu@163.com (Y.Y.); cwang@hhu.edu.cn (C.W.); xwang2014@hhu.edu.cn (X.W.); hubin_hhu@163.com (B.H.); 2 College of Environment, Hohai University, Nanjing 210098, China

**Keywords:** multiobjective reservoir operation, environmental flows, Three Gorges Reservoir, turbidity maximum zone, suspended sediment concentration, Yangtze River Estuary, improved nondominated sorting genetic algorithm III

## Abstract

The construction of multifunction reservoirs is important for flood control, agriculture irrigation, navigation, and hydropower generation, but dam construction will inevitably affect the downstream flow and sediment regimes, which can cause some environmental and ecological consequences. Therefore, this paper aims to propose a framework for assessing the multiobjective reservoir operation model based on environmental flows for sustaining the suspended sediment concentration (SSC) requirements in the turbidity maximum zone (TMZ). The Yangtze River Estuary was used as a case study. Through using an analytical model, a quantitative correlation between SSC and water flow rate was established. Then, the quantitative correlation and the SSC requirements were applied to determine the environmental flows for the estuarine TMZ. Subsequently, a multiobjective reservoir operation model was developed for the Three Gorges Reservoir (TGR), and an improved nondominated sorting genetic algorithm III based on elimination operator was applied to the model. An uncertainty analysis and a comparative analysis were used to assess the model’s performance. The results showed that the proposed multiobjective reservoir operation model can reduce ecological deficiency under wet, normal, and dry years by 33.65%, 35.95%, and 20.98%, with the corresponding hydropower generation output lost by 3.37%, 3.88%, and 2.95%, respectively. Finally, we discussed ecological satiety rates under optimized and practical operation of the TGR in wet, normal, and dry years. It indicated that the multiobjective-optimized runoff performs better at maintaining the TMZ in the Yangtze River Estuary than practical runoff. More importantly, the results can offer guidance for the management of the TGR to improve the comprehensive development and protection of the estuarine ecological environment.

## 1. Introduction

The construction and operation of multifunction reservoirs can provide water supply, agriculture irrigation, flood control, navigation, and hydropower generation [1]. The construction of dams decreased sediment load entering the estuary and sea, which would affect physical, chemical, biological, and geological processes in these areas. As a result, various serious environmental issues occurred, such as eutrophication, phytoplankton blooms, oxygen depletion, erosion of the subaqueous delta, and interference with fish migration and survival [2,3,4,5,6,7].

Estuaries, where freshwater from upstream rivers mixes with saltwater from the sea, host interactions between the lithosphere, hydrosphere, atmosphere, and biosphere. Within estuaries, there is a reach where the suspended sediment concentration (SSC) is higher than that in the adjacent waters; commonly, this region is termed the turbidity maximum zone (TMZ). The spatial–temporal variability and the behavior of TMZ were studied in many estuaries, such as the Capibaribe estuary in Brazil [8], the Loire estuary in France [9], and the Sabaki estuary in Kenya [10]. The TMZ, which migrates regularly within an estuary [11,12], plays an important ecological role in the estuary through nutrient transformation and plankton abundance [5,13], heavy metal distribution [14,15], and anadromous fish productivity [16,17].

The TMZ in the Yangtze River Estuary is well developed and characterized by the high wash-load content [18]. The mechanisms of TMZ formation in the Yangtze River Estuary include tidal asymmetry, gravitational circulation, near-bed periodic tidal resuspension, and turbulence suppression by cohesive suspension/salinity stratification [19,20]. In the Yangtze River Estuary, the TMZ is delivered by the local resuspension of the accumulated materials and bed erosion [21]. It shifts seaward in the summer and landward in the winter due to seasonal variations in runoff into the estuary [22,23]. The SSC delivered by the Yangtze River into the estuary is dominated by freshwater discharge, deforestation, land use, soil erosion, and dam impoundment [24].

Extensive anthropogenic activities, such as water withdrawals, interbasin water transfers, and dam construction, have affected flow and sediment regimes in the middle and lower Yangtze River [1,25]. After the TGR began operation in 2003, riverine sediment transport into the Yangtze River Estuary has greatly changed because of the sharp decrease in SSC and suspended sediment discharge (SSD) [23,24]. Since the Three Gorges Dam (TGD) impoundment, the reduction of river discharge has resulted in a decrease in the areas of the TMZs and changes of topographic features in all channels [26]. The TGD caused a reduction in TMZ area of 12.88% and 1.24% during spring and neap tides, respectively [25]. Between 1959–1999 and 2000–2009, the average SSC for the TMZ in Yangtze River Estuary decreased by 24.73% and the latitudinal range of the TMZ shrank inwards for 1/6 degree [27].

Because of TGR operations, the construction of new large dams, and the South-to-North Water Diversion project in the Yangtze River watershed, the sediment load into the Yangtze River Estuary will continue to decline in future decades [6]. To maintain the location and area of an estuarine TMZ, it is imperative for water resource managers to evaluate the effect of TGR operations on SSC in critical TMZ regions. In the Yangtze River Estuary, the extent and magnitude of the TMZ varies with upstream water discharge [25]. The forward shift of the TMZ was determined by runoff and tide, and the sharp decrease of the TMZ area was determined by SSD from the upstream watershed [27]. Furthermore, the location, area, and average SSC of the TMZ in the estuary were considerably changed by the TGD. Research on freshwater requirements from upstream drainages for preserving the estuarine TMZ is crucial for maintaining the estuary’s role as a filter; such research has significant theoretic value and wide potential applications.

Fang et al. [28] presented a one-dimensional numerical model for unsteady and nonuniform sediment transport and applied it to the Hongshui and Yellow Rivers in China with good results. Shi et al. [29] developed a two-dimensional horizontal numerical model of fine-sediment transport to predict fine-sediment transport processes within the TMZ at the South Channel–North Passage of the Yangtze River Estuary. Zhang et al. [30] developed a process-based sediment transport model under a wide variety of wave and current conditions. Park et al. [31] developed a three-dimensional intertidal sediment transport model to study TMZ characteristics along the main channel of the upper Chesapeake Bay. Grasso et al. [32] used a 3D process-based mixed-sediment (i.e., mud, sand, and gravel) numerical model, forced by wind, waves, tides, and river flow, to quantify the influence of the main forces (river flow, tides, and waves) on the TMZ location and mass changes. These modeling studies could accurately predict the water flow rate and suspended sediment transport processes in coastal regions when basic estuarine topography, hydrology, and sediment concentration data were sufficiently known. However, these data (especially topographic data) are difficult to obtain in many estuarine and coastal waters, and few studies have focused on the impacts of freshwater changes on position variations of the TMZ. One-dimensional analytical models for suspended sediment transport have a simple structure that can be easily programmed and requires limited data. Additionally, the simulation accuracy of the analytical model does not decrease significantly compared with that of numerical models for predicting mass and heat transfer in many studies [33,34]. Accordingly, a one-dimensional sediment transport analytical model was used in this study to assess the influence of upstream freshwater on the decrease in SSC and the shift of the TMZ in the estuary.

Conventional reservoir operation usually maximizes economic benefits, but their influences on society and the river ecosystems are often ignored [35,36,37]. Numerous methods for analysis of proper functioning of critical infrastructures have been developed so as to achieve improved safety and reliability [38]. Considering environmental sustainability and water supply reliability, a new paradigm which incorporates the deficits of environmental flow requirement into modern reservoir operations for multiobjective water resources planning and management has been developed [39,40,41]. Besides, comparative analysis of reservoir operation results and environmental flow requirements was performed to evaluate the performance of the scheduling model [42].

In this study, we first established a quantitative relationship between the SSC and water flow rate using an analytical model that was calibrated and validated by comparison with in situ hydrological and sediment concentration data. Based on the quantitative relationship and the SSC objectives, we determined the environmental flows required to sustain the SSC requirements for upper-limit cross sections of the TMZ. Then, a multiobjective reservoir operation model was developed to achieve the economic, societal, and environmental benefits simultaneously. We chose the wet, normal, and dry years as the three typical hydrological years and applied the improved nondominated sorting genetic algorithm III based on the elimination operator (NSGA-III-EO) to the model. Finally, practical operation results, multiobjective-optimized operation results, and the environmental flows were analyzed by means of an uncertainty analysis and a comparative analysis, and the results were used to formulate suggestions for water resource management in the estuary that will aid in the preservation of the TMZ.

## 2. Materials and Methods

### 2.1. Study Area and Data

The Yangtze River is the largest river in Asia in terms of length and the fourth largest in the world in terms of fluvial sediment load [18,43]. The TGR located at the upper end of the Yangtze River is the largest hydroelectric power station in the world, with maximum and minimum releases of 98,800 and 1580 m^3^/s, respectively. The operation of the TGR alters the river’s monthly flow distribution and significantly decreases the annual mean SSD [23].

**Figure 1:**
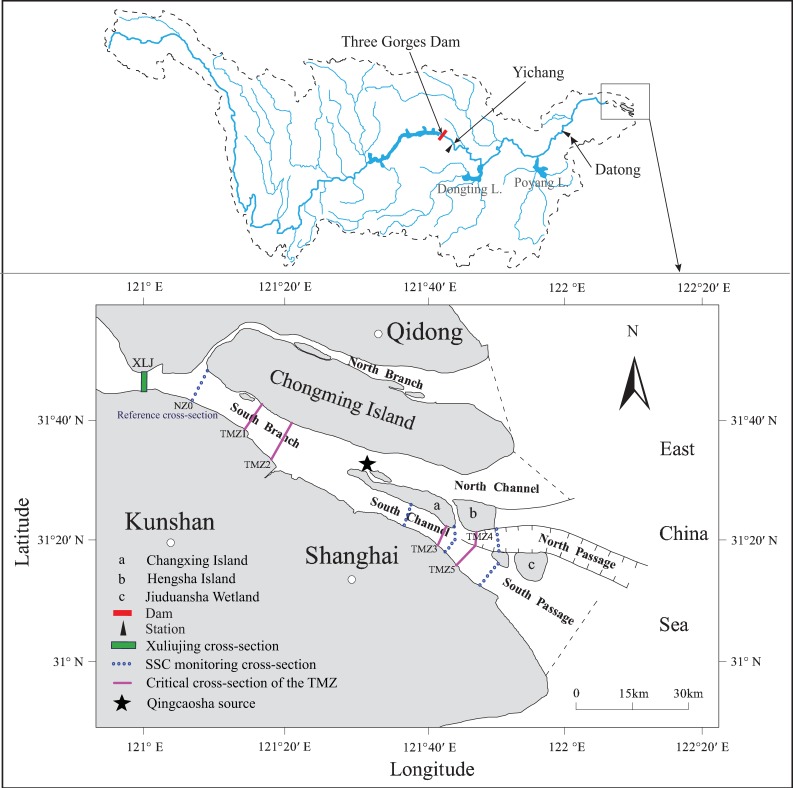
The Yangtze River Estuary (SSC refers to the suspended sediment concentration, TMZ refers to the turbidity maximum zone).

The Yangtze River Estuary is a mesotidal estuary characterized by three-order bifurcations and four outlets into the East China Sea (Figure 1). Estuarine turbidity maximums exist in the outlets of each branch, but the locations and formation mechanisms of the TMZs are different [44]. The longitudinal extension of the TMZ in the Yangtze River Estuary ranges from 25 to 46 km; the SSC in surface waters varies between 0.1 to 0.7 kg/m^3^, while the SSC in bottom waters varies between 1.0 to 8.0 kg/m^3^ [45].

The data used in this study included topography, SSC, and river discharge, as well as suspended sediment and bed material grain composition. Topography comprises the cross-sectional area, width, and water depth of the Yangtze River Estuary’s branches. The water depth was measured in situ and the width was determined using Google Earth; these values were then used to calculate cross-sectional area. The water discharge data were taken from the Hydrologic Year Book for the Yangtze River Basin. The water depth, SSC, and grain composition data were obtained from the Data Archive and Sharing Network for Resource and Environment Projects of 973 Program (http://www.973geodata.cn). The depth-averaged SSC and water depth data were simultaneous observations at hourly intervals during a spring tide, made on 13–14 February 2006 and 30–31 January 2010 for each cross section along the estuary. We took the cross-sectional hourly averaged observations as the SSC and water depth of the cross section.

### 2.2. Suspended Sediment Concentration Objectives for the TMZ

The upper boundary of the TMZ has been widely used to evaluate the location variation of the TMZ under the influence of human activities [25,27]. In this study, we used the upper limit of the TMZ during the pre-TGD period (with the corresponding critical SSC) to quantify the environmental flows needed to maintain the location of current TMZ.

The locations of TMZs vary in the different branches. Chen et al. [25] determined that the TMZ was a region with surface SSC higher than 0.9 kg/m^3^ during the spring tide. They also pointed out that there were two separate parts of the TMZ in the South Branch during spring tide, with upper boundaries located 35.32 km and 103.15 km from the Xuliujing cross section in the pre-TGD period. Accordingly, we assigned the upper boundary of the upstream part as TMZ1 in the South Branch and the upper boundaries of the downstream part as TMZ4 and TMZ5 in the North Passage and South Passage, respectively. Jiang et al. [18] determined that the general upper limit of the TMZ in the Yangtze River Estuary was approximately 121°20′ E, which we considered the equivalent of TMZ2 in this study. We used a surface SSC value higher than 0.7 kg/m^3^ to empirically indicate the TMZ [18]. Moreover, according to hydrological measurements in the estuary for the period 1959–1999, Yang et al. [27] concluded that the peak zone of SSC was 121°45′ to 122°30′; thus, the latitude 121°45′ E was considered as the upper limit of the TMZ3, with 0.70 kg/m^3^ as the critical surface SSC value. An upstream cross section of the South Branch is selected as a reference cross section (NZ0) at the landward boundary. The SSC requirements and critical cross sections for the TMZ are shown in Table 1 and Figure 1.

### 2.3. The Inflows of the TGR in Different Exceedance Probabilities

The ten-day inflow data of the TGR is uncertain within each year, so exceedance probabilities are used in this study for selecting typical hydrological years to trigger the multiobjective reservoir operation model. The annual inflow data of the TGR from 2003 to 2016 are analyzed. The inflow data less than 30%, 50%, and 70% of historical values represent a wet year, a normal year, and a dry year, respectively. The exceedance probabilities of 0% and 100% represent the maximum and minimum inflow data of the TGR, respectively. Table 2 lists the annual inflow values of the TGR corresponding to different exceedance probabilities. Practical 10-day inflow and outflow data under different exceedance probabilities can be seen in File S1. 

### 2.4. Suspended Sediment Concentration Calculation for the Alluvial Estuaries

#### 2.4.1. Modification of Shape Description of Alluvial Estuaries

The analytical solution for the sediment transport model is developed on the basis that the topography can be accurately modeled. Based on the theory developed by Savenije [46], alluvial estuaries have converging banks and their shape can be described by some exponential functions. Meanwhile, topographical changes caused by anthropogenic activities need to be considered [47,48]. The shape of alluvial estuaries needs to be corrected by the correction coefficients. Afterward, we improved the shape description of alluvial estuaries as follows:

(1) $$A=(A_{0}+A_{0}^{'})\exp\left(-\frac{x_{e}}{a_{i}+a_{i}^{'}}\right)\;\left(i=1\right),\;A=\;(A_{\left(i-1\right)}+A_{\left(i-1\right)}^{'})\exp\left(-\frac{x_{e}-x_{e\left(i-1\right)}}{a_{i}+a_{i}^{'}}\right)\;\left(i>1\right)\;$$

(2) $$B=(B_{0}+B_{0}^{'})\exp\left(-\frac{x_{e}}{b_{i}+b_{i}^{'}}\right)\;\left(i=1\right),\;B=(B_{\left(i-1\right)}+B_{\left(i-1\right)}^{'})\exp\left(-\frac{x_{e}-x_{e\left(i-1\right)}}{b_{i}+b_{i}^{'}}\right)\;\left(i>1\right)\;$$

(3) $$h=(h_{0}+h_{0}^{'})\exp\left(x_{e}\left(\frac{a_{i}-b_{i}}{a_{i}b_{i}}+h_{i}^{'}\right)\right)\;\left(i=1\right),\;h=(h_{\left(i-1\right)}+h_{\left(i-1\right)}^{'})\exp(\left(x_{e}-x_{e\left(i-1\right)}\right)\left(\frac{a_{i}-b_{i}}{a_{i}b_{i}}+h_{i}^{'})\right)\;\left(i>1\right)\;$$

where A, B, and h are the cross-sectional area, width, and depth away from the estuary mouth $x_{e}$ in kilometers, respectively; $A_{0}$, $B_{0}$, and $h_{0}$ are the cross-sectional area, width, and depth at the mouth; i represents the reach number of the channels—the exponential functions would be applied to describe the geometry of a single channel estuary when i=1, and that can be applied to a multiple-reaches estuary when i>1; $x_{e\left(i-1\right)}$ is the length of the (i−1)th reach; $A_{\left(i-1\right)}$, $B_{\left(i-1\right)}$, and $h_{\left(i-1\right)}$ are the cross-sectional area, width, and depth for the downstream section of the ith reach; and $a_{i}$ and $b_{i}$ represent the convergence length of the area and width for each reach, respectively. $A_{0}^{'}$, $A_{\left(i-1\right)}^{'}$, $B_{0}^{'}$,$\;B_{\left(i-1\right)}^{'}$, $h_{0}^{'}$, $h_{\left(i-1\right)}^{'}$, $a_{i}^{'}$, $b_{i}^{'}$, and $h_{i}^{'}$ are correction coefficients.

#### 2.4.2. Analytical Solution for the Steady Nonuniform and Nonequilibrium Sediment Transport Model

The erosion and deposition of fluvial sediments constitute a dynamic equilibrium in alluvial estuaries [46]. The theory for this model was proposed by Han [49]_ENREF_42: when sediment transport is in nonequilibrium, the sediment concentration and the gradations of suspended load and bed load change along the river.

For a system mixing water and sand, the continuity equation and momentum equation can be described as

(4) $$\;\frac{\partial A}{\partial t}+\frac{\partial Q}{\partial x}=0\;$$

(5) $$\;\frac{\partial Q}{\partial t}+\frac{\partial }{\partial x}\left(\frac{Q^{2}}{A}\right)+gA\frac{\partial Z}{\partial x}+gA\frac{Q\left|Q\right|}{K^{2}}=0\;$$

where x and t are spatial and temporal variables, Q is the discharge, A is the cross-sectional area, Z is the water surface elevation, g is the gravitational acceleration, and K is the runoff modulus.

Because convective motion is the major process in water and sediment transportation, the influence of the diffusion term can be ignored, leading to the following continuity equation for sediment transportation:

(6) $$\;\frac{\partial \left(\mathrm{AS}\right)}{\partial t}+\frac{\partial \left(QS\right)}{\partial x}+\rho^{\prime }\frac{\partial A^{\prime }}{\partial t}=0\;$$

where S is the cross-section-averaged sediment concentration, $\rho^{\prime }$ is the dry density of sediment, and $A^{\prime }$ is the erosion or deposition area. The study of total erosion or deposition $\rho^{\prime }\frac{\partial A^{\prime }}{\partial t}$ can be categorized as total sediment transport modeling, suspended load modeling, and bed load modeling. Traditionally, nonequilibrium transport of suspended load is adopted for calculations pertaining to unsaturated sediment transport:

(7) $$\;\rho^{\prime }\frac{\partial A^{\prime }}{\partial t}=\alpha \omega B\left(S-S_{*}\right)\;$$

where$\;S_{*}$ is the cross-section-averaged sediment transport capacity, ω is the sediment settling velocity, α is the recovery coefficient relating to adaptation from a nonequilibrium state to the equilibrium state, and B is the channel width.

A derivation process for the steady one-dimensional nonuniform suspended sediment nonequilibrium sediment transport model can be found in File S2.

The suspended sediment transport model can be expressed as follows:

(8) $$\;S=S_{*}+\left(S_{0}-S_{*0}\right)\cdot e^{-\frac{\alpha \omega BL}{Q}}+\frac{Q}{\alpha \omega BL}\cdot \left(S_{*0}-S_{*}\right)\cdot \left(1-e^{-\frac{\alpha \omega BL}{Q}}\right)\;$$

where $S_{0}$ and $S_{*0}$ are the cross-section-averaged sediment concentration and transport capacity of the upstream section, respectively, and L is the length of the river reach.

#### 2.4.3. Gradations of Suspended Load and Its Variability along the River

If the nonuniform sediment particles are separated into n classes, the particle size and settling velocity of each class is $D_{j}$ and $\omega_{j}$ (j=1,2,⋯,n), respectively:

(9) $$\;S_{j}=P_{j}S,\;S_{*j}=P_{*j}S_{*}\;$$

where $P_{j}$ and $P_{*j}$ are the gradation of suspended load concentration and the sediment transport capacity, respectively, and $S_{j}$ and $S_{*j}$ are the sediment concentration and capacity of the ith class sediment, respectively. $P_{*j}$ is assumed to equal the gradation of the suspended load concentration [50], thus $P_{*j}=P_{j}$.

(1) In the case of erosion, the gradation of suspended load is derived as

(10) $$\;P_{j}=\frac{P_{0j}-\lambda P_{j}^{*}}{1-\lambda }\;$$

where $P_{0j}$ is the initial gradation of suspended sediment, λ is the erosion ratio (denoted as $\lambda =\frac{S_{0}-S}{S_{0}}$), and $P_{j}^{*}$ is the alimentative gradation of suspended load from bed material, defined as

(11) $$\;P_{j}^{*}=P_{b0j}\frac{1-{\left(1-\lambda^{*}\right)}^{{\left(\frac{\omega_{zh}}{\omega_{j}}\right)}^{\beta }}}{\lambda^{*}}\;$$

where $\lambda^{*}$ is the thickness ratio of the eroded subsurface layer and the mixing layer, $P_{b0j}$ is the initial gradation of bed material, β is a revised index (often assumed to be 0.75 in natural river courses), and $\omega_{zh}$ is the comprehensive effective settling velocity of the sediment, satisfying the following equation determined by $\overset{n}{\underset{j=1}{\sum }}P_{j}^{*}\;=\;1$. Thus,

(12) $$\;\frac{\sum_{j=1}^{n}P_{b0j}\left[1-{\left(1-\lambda^{*}\right)}^{{\left(\frac{\omega_{zh}}{\omega_{j}}\right)}^{\beta }}\right]}{\lambda^{*}}=1\;$$

(2) In depositional situations, the gradation of the suspended load is expressed as

(13) $$\;P_{j}=P_{0j}\frac{{\left(1-\lambda \right)}^{{\left(\frac{\omega_{j}}{\omega_{zh}}\right)}^{\beta }}}{1-\lambda }\;$$

where λ is the deposition ratio, and $\omega_{zh}$ must satisfy

(14) $$\;\frac{\sum_{j=1}^{n}P_{0j}{\left(1-\lambda \right)}^{{\left(\frac{\omega_{j}}{\omega_{zh}}\right)}^{\beta }}}{1-\lambda }=1\;$$

#### 2.4.4. Sediment Settling Velocity

The settling velocity $\omega_{j}$ for each size class can be computed individually (File S3).

The comprehensive settling velocity of nonuniform sediment $\omega_{*}$ is formulated as follows:

(15) $$\;\omega_{*}={\left(\overset{n}{\underset{j=1}{\sum }}P_{j}\omega_{j}^{m}\right)}^{\frac{1}{m}}\;$$

where m represents the index of sediment transport capacity.

#### 2.4.5. Average Sediment Transport Capacity of the Cross-Sectional Area

Han [50] presented a sediment transport capacity formula for suspended load:

(16) $$\;S_{*}=k\gamma_{s}{\left[\frac{\gamma U^{3}}{\left(\gamma_{s}-\gamma \right)gH\omega_{*}}\right]}^{m}\;$$

where k is the coefficient of sediment transport capacity, H is the average depth of water, and U is the mean velocity of the cross section.

### 2.5. Multiobjective Reservoir Operation Model Development

#### 2.5.1. Objective Function

(1) Economic benefit objective

The economy objective function is the maximum benefit of hydropower generation, which is derived from a previous work [51].

(17) $$\;F=max\overset{T}{\underset{t=1}{\sum }}k\alpha *q_{t}*h_{t}*\tau_{t}\;$$

(18) $$\;h_{t}=z_{up}\left(q_{t}\right)-z_{down}\left(q_{t}\right)-z_{loss}\;$$

(19) $$\;z_{up}\left(q_{t}\right)=78.65+\sqrt{23.288*\left[V_{t}+\left(I_{t}-q_{t}\right)*\tau_{t}\right]+127.225}\;$$

(20) $$\;z_{down}\left(q_{t}\right)=62.57917+9.84891\times 10^{-5}*q_{t}+1.42007\times 10^{-9}*q_{t}^{2}\;$$

(21) $$\;z_{loss}=\{\begin{array}{ll} 1\times 10^{-4}*I_{t} & 5000<I_{t}\leq 10,000\\ 1\times 10^{-5}*I_{t}+0.9 & 10,000<I_{t}\leq 60,000 \end{array}\;$$

where F is the maximum output of hydropower generation (kW·h); k is the price of hydropower (0.2506 RMB/kW·h); α is the output factor of power generation and α = 9.81 [52]; $q_{t}$ is the water outflow rate in the tth time period $\left(m^{3}/s\right)$; $h_{t}$ is the reservoir average water head at time period t (m); $\tau_{t}$ is the duration in the ith dispatching period (s), and T denotes the whole schedule period count within a year: T=36; $z_{up}\left(q_{t}\right)$ represents the relationship between water level and water discharge; $z_{down}\left(q_{t}\right)$ represents the relationship between tail water level and water discharge; $z_{loss}$ denotes the head loss; $V_{t}$ is the volume of reservoir storage at the beginning of the tth period $\left(m^{3}\right)$; and $I_{t}$ is the reservoir inflow in the tth period $\left(m^{3}/s\right)$.

(2) Social benefit objective

The minimum shortage of the socioeconomic water requirement in the TGR area is considered as the social benefit objective function:

(22) $$\;S=min\left|\overset{T}{\underset{t=1}{\sum }}\;(D_{t}-Q_{t}-q_{t}*\tau_{t})\;\right|\;$$

where $D_{t}$ and $Q_{t}$ represent the socioeconomic water requirement and water storage volume of the reservoir region over the tth period $\left(m^{3}\right)$, respectively.

(3) Ecological objective

The ecological objective is the minimum shortage of water demand that conserves the drinking-water source:

(23) $$\;E=min\overset{T}{\underset{t=1}{\sum }}max\left[0,\left(q_{eco}-q_{t}\right)\times \tau_{t}\right]\;$$

where $q_{eco}$ is the minimum water demand for preventing seawater spill over into the drinking-water source.

#### 2.5.2. Constraints

The multiobjective ecological reservoir operation is subjected to the following constraints:

The water balance equation:

(24) $$\;V_{t+1}=V_{t}+\left(I_{t}-q_{t}\right)*\tau_{t}-EP_{t}\;$$

where $EP_{t}$ is the sum of seepage and evaporation from the reservoir in the period t $\left(m^{3}\right)$.

Flood control discharge limits:

(25) $$\;q_{t}\leq q_{F}\;$$

Shipping discharge limits:

(26) $$\;q_{t}\geq q_{S}\;$$

Reservoir discharge limits:

(27) $$\;Q_{t,min}\leq q_{t}\times \tau_{t}\leq Q_{t,max}\;$$

where $Q_{t,min}$ and $Q_{t,max}$ are the minimum and maximum water discharges of the reservoir over time period t, respectively$\;(m^{3})$.

Reservoir storage volume limits:

(28) $$\;V_{t,min}\leq V_{t}\leq V_{t,max}\;$$

Non-negative constraint:

(29) $$\;q_{t}\geq 0\;$$

The calculations were simplified by assuming that the seepage and evaporation of the reservoir was 0. The socioeconomic water demand in the Yibin–Yichang section of the Yangtze River is predicted to be $1.0058\times 10^{10}$ m^3^ in 2020, and its annual distribution was considered as the socioeconomic water demand in the reservoir region in each time period [1]. The water storage volume of the reservoir in each time period t was obtained by the annual distribution of the average storage volume of the TGR. The Qingcaosha water source located to the east of Changxing Island is the third largest water source for Shanghai, and it is easily affected by saltwater spilling over from the East China Sea. The protection of this water source is imperative and has been selected as a key ecological objective. The minimum water demand of the TGR outflow needed to ensure the optimal salinity of the Qingcaosha water source is 10,555 m^3^/s [1,53]. Thus, the value of $q_{eco}=10,555$ m^3^/s was used in the ecological objective function (Equation (23)).

The scheduling scheme of the TGR is to increase reservoir discharge by 1000–2000 $m^{3}/s$ between January and May. During mid-to-late September, immediately before the storage period, the reservoir discharge is kept above 10,000 $m^{3}/s\;$to ensure flood control safety. In October, the storage period, the reservoir discharge is kept at a minimum of 6500 $m^{3}/s$ and water discharge is decreased by 4000 $m^{3}/s$ [54]. The water discharge of the reservoir required to ensure flood control was set to 35,000 $m^{3}/s$ [51]. When the navigable condition of the TGR meets the water discharge of [5000, 56,700] $m^{3}/s$, the ship can pass through the TGR smoothly [51]. The minimum and maximum capacities of the reservoir were set to $V_{t,min}=1.175\times 10^{10}{\;m}^{3}$ and $V_{t,max}=3.93\times 10^{10}{\;m}^{3}$, respectively.

#### 2.5.3. Improved Nondominated Sorting Genetic Algorithm III Based on the Elimination Operator (NSGA-III-EO)

The NSGA-III algorithm proposed by Deb and Jain [55] is similar to the original NSGA-II [56]. The significant breakthroughs of NSGA-III are the decomposition-based idea and the selection mechanism. Based on the above, Bi and Wang [57] proposed an improved nondominated sorting genetic algorithm III (NSGA-III-EO) with the elimination operator to create new individuals.

General framework of NSGA-III-EO:

Input:Multiobjective optimization problem;  a termination criterion;  N: the number of population (size) considered in NSGA-III-EO;  $Z=\left\{\lambda^{1},\;\lambda^{2},\ldots ,\lambda^{N}\right\}$: a set of N reference points;Output:  Approximation to the Pareto optimal set and the Pareto optimal objective vectors.Step 1: Initialization:Generate reference points *Z*;Generate an initial population $P_{0}$. Set i=0.While the termination criterion is not met, generate a new offspring $Q_{i}$ from parents $P_{i}$ by applying binary crossover and polynomial mutation, and then combine parent and child populations, and set $R_{i}=P_{i}\cup Q_{i}$;Nondominated sorting of $R_{i}$, set $\left(F_{1},F_{2},\cdots \right)$ which are all PF of $R_{i}$, and set $S_{i}=\emptyset ,\;k=1$;Step 2: Update:Include all nondominated fronts in the new population $S_{i}$, set k=k+1 and $S_{i}=S_{i}\cup F_{k}$, until the size of new population is equal to or for the first time becomes larger than the size of the parent population, thus $\left|S_{i}\right|\geq N$, and the last front to be included is $F_{l}=F_{k}$;Step 3: Termination criterion:If $\left|S_{i}\right|=N$, then $P_{i+1}=S_{i},\;$break. Output P=PS∪PF; or else normalize the individuals in $S_{i}$ with reference points Z, then eliminate individuals from $S_{i}$: $K=\left|S_{i}\right|-N$;Associate the normalized individual in $S_{i}$ according to the perpendicular distance of each individual of $S_{t}$ from each of the $\lambda^{i}$;The worst individual whose associated reference point has the maximum niche count is eliminated from $S_{i}$, then i=i+1 and go to Step 2.

#### 2.5.4. The Quantitative Relationships of Water Flow Rate along the Yangtze River

Yu et al. [58] developed regression models for assessing the impact of the TGR impoundment on the downstream water flow rate. The relationship of water flow rate between the Yichang and Datong hydrologic station is shown in Equation (30):

(30)  y=1.153x+11744.563 

where x is the daily water flow rate $\left(m^{3}/s\right)$ at the Yichang station and y is the daily water flow rate $\left(m^{3}/s\right)$ with a 6-day lag at the Datong station.

The relationship of water flow rate between the TGR outflow and the Yichang station is shown in Equation (31):

(31)  y=1.032x−180.614 

where x is the daily TGR outflow $\left(m^{3}/s\right)$ and y is the daily river flow rate $\left(m^{3}/s\right)$ without a time lag at the Yichang station.

## 3. Results and Discussion

### 3.1. Calibration of the Steady Nonuniform and Nonequilibrium Sediment Transport Model for the Yangtze River Estuary

**Figure 2:**
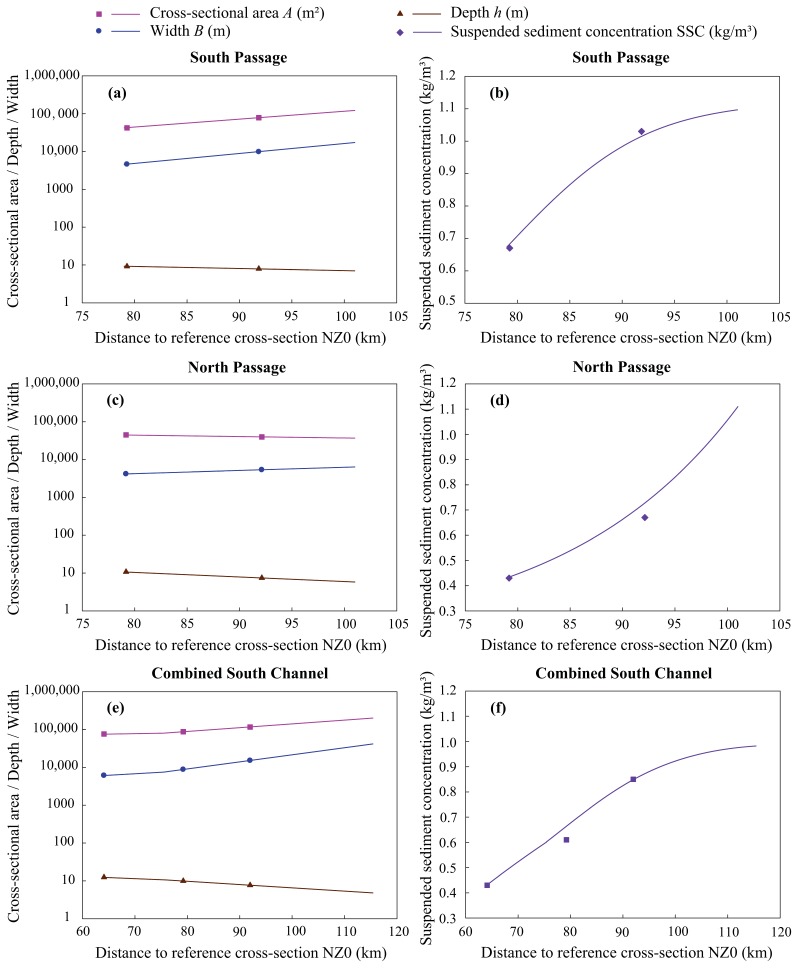
The computed topography and SSC distribution for the South Passage (**a**,**b**), North Passage (**c**,**d**), and the combined South Channel (**e**,**f**) on 13–14 February 2006, compared to observations. (Scatters represent observations, and the drawn lines represent the simulated values).

Although we sourced the topographical parameters of the Yangtze River Estuary’s branches from Zhang et al. [59], which is listed in Supplementary Materials Table S1, we obtained the topographical correction coefficients for the estuary by calibration. We used two approaches to calculate the SSC distribution. First, we calculated the SSC distribution for the signal channels of the South and North Passages. Second, we calculated the SSC distribution for the combined South Channel; the width and area of the combined channel were the sum of the widths and areas of the separate channels. The combined water depth was the ratio of combined area to combined width. The SSC of the combined channel was thus a weighted average of the separate channels, in which the cross-sectional area of each separate channel was taken as the weight. Based on the above topographical parameters and SSC computational approaches, the topography and SSC distribution for the South Passage, North Passage, and the combined South Channel have been calibrated using data observed on 13–14 February 2006 during the spring tide. The calibrated results are presented in Figure 2. The computed cross-sectional area, width, water depth, and SSC fit the observed data well, both for the separate and combined channels of the estuary’s branches.

River discharge is an important parameter in the calibration process of SSC distribution. The freshwater in a multichannel estuary is separated into different branches, and it is difficult to observe the discharge from separate channels because of the tidal influence. Therefore, we considered the river discharge observed at the Datong Hydrometric Station as the river discharge into the Yangtze Estuary.

### 3.2. Validation of the Steady Nonuniform and Nonequilibrium Sediment Transport Model for the Yangtze River Estuary

**Figure 3:**
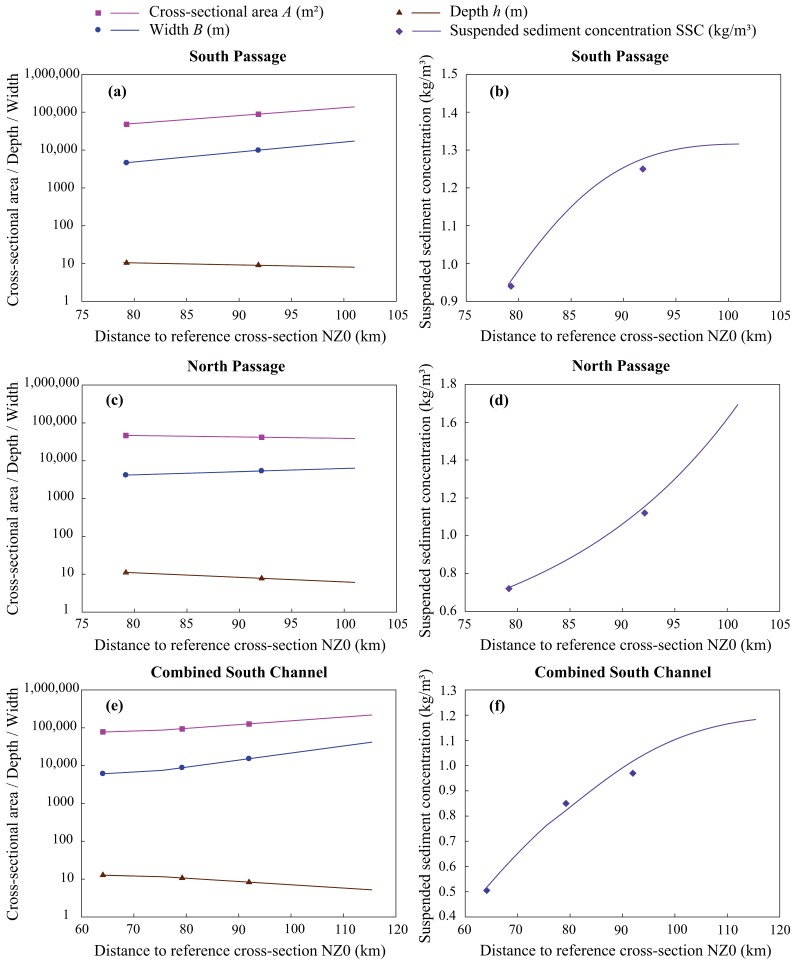
The computed topography and SSC distribution for the South Passage (**a**,**b**), North Passage (**c**,**d**), and the combined South Channel (**e**,**f**) on 30–31 January 2010, compared to observations. (Scatters represent observations, and the drawn lines represent the simulated values).

The topographical and SSC data measured on 30–31 January 2010 during the spring tide from the same six cross sections of the Yangtze River Estuary as calibration values were used to validate the suspended sediment transport model. Figure 3 shows that the topography and SSC distribution curves fit the monitoring data well. Therefore, the suspended sediment transport model can be used to obtain environmental flows for SSC requirements.

SSC distributions in the Yangtze River Estuary are somewhat complicated because there are three sources of suspended sediment: from sediment transport of upstream river discharge, from in situ suspended sediment–bed sediment exchange and the resuspension process, and from the sea. As shown by the distribution curves in Figure 3b,d,f, the increasing rate of SSC in the North Passage is higher than that in the South Passage and the combined South Channel during the spring tide, which is not only due to the south-leading jetty in the middle of the North Passage containing a large amount of overlapping sediments, but is also related to the concentrated parts of overbank sediments and back siltation [27,60]. Meanwhile, the reduced sediment flux from the upstream watershed and coarsening of the bed sediments will lead to the reduction of the SSC downstream [27]. These situations require more detailed future research using specific observations.

### 3.3. Environmental Flow Calculation for Estuarine TMZ

**Figure 4:**
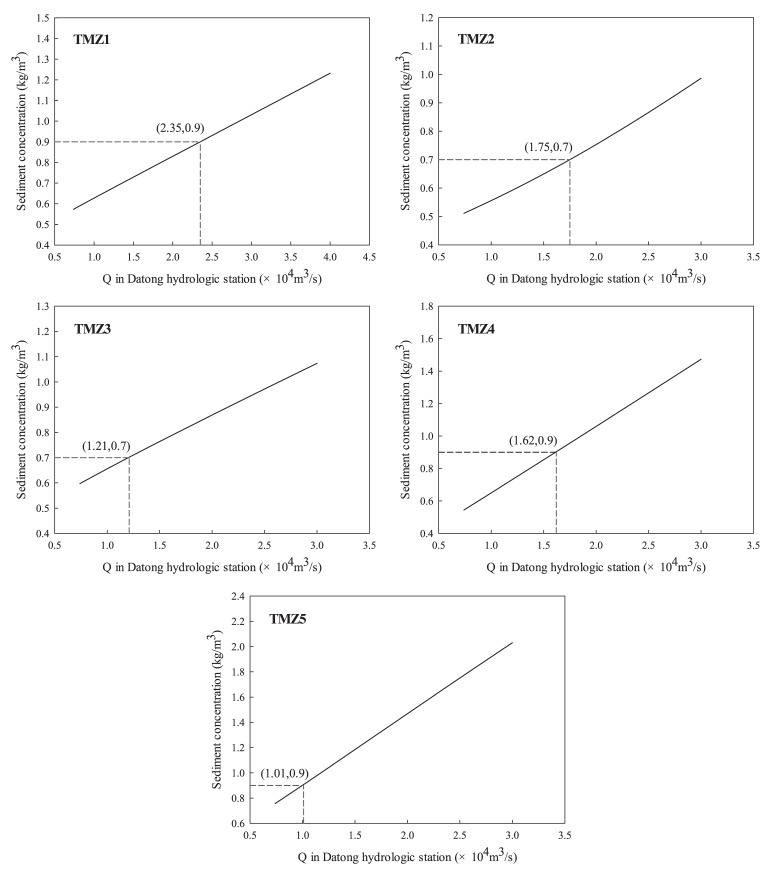
Relationships between water flow rate and SSC at the upper-limit cross sections of the TMZ (Q refers to the river discharge).

The relationship between the water flow rate recorded at the Datong control station and SSC objectives in the five critical upper-limit cross sections of the TMZ were built to obtain the environmental flows for maintaining the location of the estuarine TMZ (see Figure 4). The corresponding environmental flows were listed in Table 3.

### 3.4. Uncertainty Analysis of Objective Values between Practical Operation and Multiobjective-Optimized Reservoir Operation

**Figure 5:**
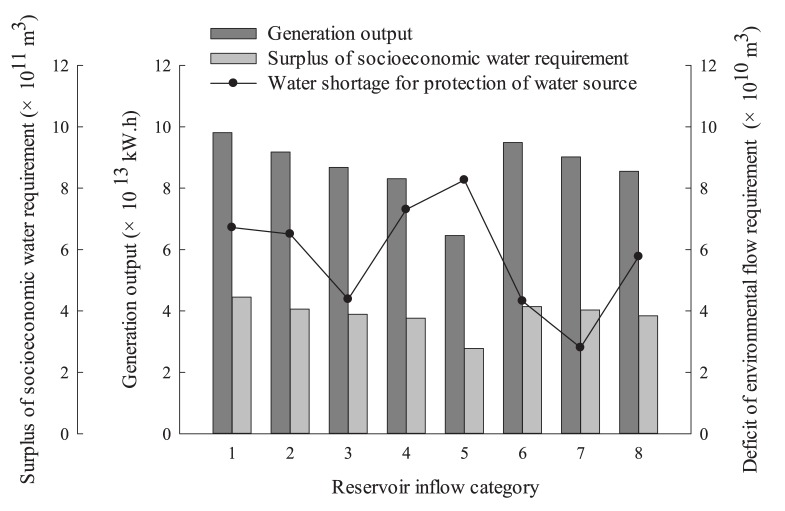
Objective values of the practical operation and multiobjective optimal operation of the TGR. (The reservoir inflow categories from 1 to 5 correspond to the actual operation under exceedance probability from 0 to 100% in Table 2, and reservoir inflow categories 6, 7, and 8 correspond to the optimal operation under wet, normal, and dry years, respectively).

The objective function values of practical operation with inflow of the TGR in different exceedance probabilities in Table 2 are all chosen to compare with the multiobjective-optimized objective function values for the uncertainty analysis. The results are shown in Figure 5.

It can be seen from Figure 5 that the hydropower generation output and surplus of socioeconomic water requirement of the practical operation exhibit a declining trend with the exceedance probability increasing from 0 to 100%. When the exceedance probability is below 50%, namely, in categories 1–3, the water shortages for protection of the water source are significantly less than the other exceedance probability conditions. This indicates that the practical operation of the reservoir cannot balance the economic, social, and environmental benefits. However, the objective values under multiobjective-optimized operation indicate positive performance. Compared with practical operation, the shortage of water demand for protecting the drinking-water source under wet, normal, and dry years (the exceedance probabilities are 30%, 50%, and 70%, respectively) with multiobjective-optimized operation has declined by 33.65%, 35.95%, and 20.98%, respectively, at the cost of hydropower generation output lost by 3.37%, 3.88%, and 2.95%, respectively. This indicates that water shortage for protection of the water source reduces significantly using the multiobjective-optimized reservoir operation model presented in this study.

### 3.5. Comparative Analysis of Practical Operation and Multiobjective-Optimized Operation Results

In order to analyze the optimization performance of the multiobjective reservoir operation, the practical operation and multiobjective-optimized operation results on the downstream Datong cross section and the environmental flows requirement for five critical cross sections of the TMZ in the three typical hydrological years are chosen for a comparative analysis. The optimization performance of reservoir operation under different environmental flow requirements is discrepant, so reservoir operation results should meet different environmental flow requirements. Ecological satiety rate is proposed here to evaluate the optimization performance under different environmental flows. It can be calculated as follows:

(32) $$\;\frac{\sum_{j=1}^{5}W_{j}}{5\times T}\times 100\%\;$$

where $W_{j}$ is the number of time periods during which the water discharge is larger than the environmental flow requirement for the jth TMZ, and T is the whole schedule period count.

**Figure 6:**
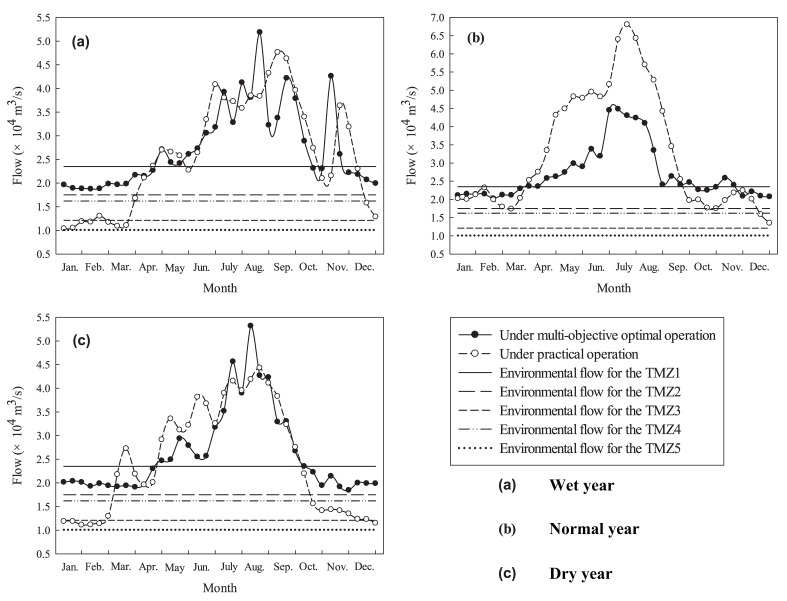
Practical operation and multiobjective-optimized operation results on the downstream Datong cross section and the environmental flows for 5 critical cross sections of the TMZ in the three typical years.

Figure 6 shows the results under both practical and optimized operation at the downstream Datong cross section and environmental flow requirements for five different critical cross sections of the TMZ in the three hydrological years. The water discharges in practical operation were obtained by observed values at the Datong station in wet, normal, and dry years, respectively. It illustrates that the water discharge after multiobjective optimization could satisfy the environmental flow requirements of TMZ2, TMZ3, TMZ4, and TMZ5 for the full year of the three typical years, while the water discharge under practical operation could only meet the environmental flow requirements of TMZ5 for the full year of the wet and dry years (shown in Figure 6a,c). Figure 6b shows that the water discharge under practical operation can meet the environmental flow requirements of TMZ3 and TMZ5 for all of the normal year. Figure 6 shows that the proposed strategy of reservoir multiobjective optimization operation can significantly increase water discharges in the dry season (from December to April) for maintaining the environmental flow requirements of the TMZ. The ecological satiety rates considering environmental flow requirements for five different critical cross sections of the TMZ under practical operation of the TGR in wet, normal, and dry years are 75.56%, 87.22%, and 70.56%, respectively. Based on the multiobjective-optimized operation of the TGR, the ecological satiety rates considering environmental flow requirements for five different critical cross sections of the TMZ in wet, normal, and dry years are 90.56%, 91.67%, and 89.44%, respectively. The ecological satiety rate is larger for the normal year than those for wet and dry years in both the practical and optimized operation results, which means that the ecological influence of the reservoir operation is smaller in the normal year. These results indicate that the developed multiobjective reservoir operation model is beneficial for maintaining the TMZ in the Yangtze River Estuary.

In the past 60 years, a number of high dams with large reservoirs have been built in the Yangtze River basin, including the TGD. As a result, large amounts of sediment discharge are trapped behind these dams. Decreasing SSD from the river basin to the estuary has affected the TMZ area and its distribution. During the past 30 years, the average area of the TMZ in the Yangtze River Estuary declined by 23%, corresponding to a 77% reduction in sediment load from the basin [18]. The SSC in several measurement points in the Yangtze River Estuary did not show an obvious drop compared with previous measurements [27,61]. The above results show a possible explanation, in that the TMZ depends upon sediments not only from the upstream basin, but also from local sediment resuspension and from the sea. That is, the proposed multiobjective reservoir operational strategy for the TGR is beneficial for maintaining the TMZ. Additionally, further study is needed on how the estuarine SSC can be calculated with consideration of a dramatic drop of the SSC released from the dam.

## 4. Conclusions

We formulated a multiobjective reservoir operation model to assess the effect of TGR operations on the locational variation of the TMZ in the Yangtze River Estuary. The relationship between water flow rate and SSC at five critical cross sections was established using an analytical model. Based on the SSC objectives for different locations in the branches, we determined the environmental flows for the Yangtze River Estuary. The multiple objective functions of the operation model, including maximizing benefit of hydropower generation, minimizing shortage of the socioeconomic water requirement in the reservoir area, and minimizing deficit between water discharge and the water demand for protecting the drinking-water source, have been considered simultaneously to obtain comprehensive benefit with respect to the economy, society, and environment. In addition, the NSGA-III-EO was applied to the model. It is believed that the proposed method is flexible for use in assessing the impact of other reservoir operation schemes on estuarial TMZ in other alluvial estuaries, using location, area, or mass changes of TMZs in these assessments.

The results show that the practical operating scheme of the TGR under the three hydrological years is sufficient for the socioeconomic water requirement of the reservoir area; however, the released flows from the reservoir cannot satisfy the environmental flow requirements for certain TMZ locations in the dry season. After the optimized operation of the TGR, the water shortage for protection of the water source under wet, normal, and dry years have declined by 33.65%, 35.95%, and 20.98%, with the corresponding hydropower generation output lost by 3.37%, 3.88%, and 2.95%, respectively. The ecological satiety rates under practical operation of the TGR in wet, normal, and dry years are 75.56%, 87.22%, and 70.56%, respectively. Based on the multiobjective-optimized operation of the TGR, the ecological satiety rates in wet, normal, and dry years are 90.56%, 91.67%, and 89.44%, respectively. The recommended multiobjective optimal operating scheme offers good performance for protecting the water source and sustaining the suspended sediment concentration for the TMZ simultaneously at the cost of power generation.

These results can provide important guidance for developing and utilizing fishery resources and comprehensively developing and protecting the estuarine environment. In addition, more measurements should be undertaken by boat in different tributaries in wet, normal, and dry years during spring, mean, and neap tides, to potentially improve the accuracy and verification of the sediment transport model. The decreasing sediment load released from the TGR also needs to be considered to clarify the influence of the TGD on the estuarine TMZ in further work.

## Figures and Tables

**Table 1:** SSC objectives for the upper limits of the five TMZs in the Yangtze River Estuary.

Branch Channel	Critical Cross Section	Corresponding Period	Critical SSC for the TMZ (kg/m^3^)	Reference	Distance to NZ0 (km)
South Branch	TMZ1 (35.32 km from Xuliujing)	Pre-TGD (before 1 June 2003)	0.9	[25]	17.72
South Branch	TMZ2 (121°20′ E)	In 1981 and 1995	0.7	[18]	25.97
South Channel	TMZ3 (121°45′ E)	From 1959 to 1999	0.7	[27]	76.55
North Passage	TMZ4 (103.15 km from Xuliujing)	Pre-TGD (before 1 June 2003)	0.9	[25]	85.55
South Passage	TMZ5 (103.15 km from Xuliujing)	Pre-TGD (before 1 June 2003)	0.9	[25]	85.55

**Table 2:** The annual inflow values of the TGR in different exceedance probabilities.

Exceedance Probabilities (%)	Annual Inflow of the TGR (×10^8^ m^3^/a)
0	4559.16
30	4252.89
50	4060.56
70	3869.50
100	2979.84

**Table 3:** Environmental flow requirements for the five critical cross sections of the TMZ ($\times 10^{4}{\;m}^{3}/s$).

Critical Cross Section	Environmental Flows
TMZ1	2.35
TMZ2	1.75
TMZ3	1.21
TMZ4	1.62
TMZ5	1.01

## Supplementary Materials

The following are available online at http://www.mdpi.com/1660-4601/15/10/2118/s1, File S1: Original measurement data and calibrated parameters for the suspended sediment concentration calculation for the alluvial estuaries and the multi-objective reservoir operation model, File S2: Derivation process of the steady one-dimensional non-uniform suspended sediment non-equilibrium sediment transport model, File S3: The sediment settling velocity formula for each size class, Table S1: Topographical parameters of the Yangtze River Estuary branches.
