# Supplementary material for: Assessment of the Multi-Objective Reservoir Operation for Maintaining the Turbidity Maximum Zone in the Yangtze River Estuary
Source: Int J Environ Res Public Health. 2018 Sep 26;15(10):2118. doi: 10.3390/ijerph15102118 (PMC6210024; doi:10.3390/ijerph15102118)
Supplement: Supplementary file 1 [file ijerph-15-02118-s001.zip › Table S1.docx]

**Table S1** Topographical parameters of the Yangtze River Estuary branches [52]

| Channel | *A*_0_ (m^2^) | *B*_0_ (m^2^) | *a*_1_ (km) | *b*_1_ (km) | *A*_1_ (m^2^) | *B*_1_ (m^2^) | *a*_2_ (km) | *b*_2_ (km) | $x_{e1}$(km) |
| --- | --- | --- | --- | --- | --- | --- | --- | --- | --- |
| North Branch | 140000 | 21000 | 6.5 | 7 | 64.872 | 10280 | 24 | 32 | 5 |
| North Channel | 122000 | 18000 | 45 | 24 | 81.779 | 8880 | 150 | 100 | 18 |
| North Passage | 65000 | 7500 | 50 | 60 |  |  |  |  |  |
| South Passage | 160000 | 24000 | 30 | 26 |  |  |  |  |  |
| Combined South Channel | 210000 | 31300 | 38 | 28 | 73.294 | 7501 | 150 | 100 | 40 |
| Combined South Branch | 350000 | 50000 | 42 | 32 | 155.774 | 17280 | 140 | 110 | 34 |
