# Supplementary material for: Assessment of the Multi-Objective Reservoir Operation for Maintaining the Turbidity Maximum Zone in the Yangtze River Estuary
Source: Int J Environ Res Public Health. 2018 Sep 26;15(10):2118. doi: 10.3390/ijerph15102118 (PMC6210024; doi:10.3390/ijerph15102118)
Supplement: Supplementary file 1 [file ijerph-15-02118-s001.zip › File S1.docx]

**File S1. Derivation process of the steady one-dimensional non-uniform suspended sediment non-equilibrium sediment transport model**

Combining equations (6) and (7), the governing equation for the non-equilibrium transport of the non-uniform suspended load can be expressed as follows:

$\frac{\partial(AS)}{\partial t}+\frac{\partial(QS)}{\partial x}+\alpha\omega B(S-S_{*})=0$ (33)

Substituting continuum equation (4) into equation (33) leads to the governing equation for the non-equilibrium transport of the non-uniform suspended load under steady flow conditions, namely let $\frac{\partial}{\partial t}=0$:

$Q\cdot\frac{\partial S}{\partial x}+\alpha\omega B\left( S-S_{*} \right)=0$ (34)

The general solution of equation (34) is

$S-S_{*}=e^{-\int\left( \frac{\alpha\omega B}{Q} \right)dx}\cdot\left[ \int\left( \left( -1 \right)\cdot\frac{dS_{*}}{dx}\cdot e^{\int\left( \frac{\alpha\omega B}{Q} \right)dx} \right)dx+c \right]$ (35)

where c is the integration constant; considering the initial conditions as $x=0$, $S=S_{0}$, and $S_{*}=S_{*0}$, substituting $x=0$ into equation (35) yields

$c=S_{0}-S_{*0}$ (36)

Substituting equation (36) into equation (35), the sediment concentration formula can be obtained:

$S=S_{*}+e^{-\frac{\alpha\omega BL}{Q}}\left[ S_{0}-S_{*0}-\int_{0}^{L} \left( e^{\frac{\alpha\omega Bx}{Q}}\cdot\frac{dS_{*}}{dx} \right)dx \right]$ (37)

where $S_{0}$ and $S_{*0}$ are the cross-section-averaged sediment concentration and transport capacity of the upstream section, respectively, and $L$ is the length of the river reach. The further integral needs the expression of the formula $\frac{dS_{*}}{dx}$, as the sediment transport capacity is assumed as a linear change along the river. Thus, $\frac{dS_{*}}{dx}=-\frac{S_{*0}-S_{*}}{L}$; substituting this function into equation (37) yields

$$S=S_{*}+(S_{0}-S_{*0})\cdot e^{-\frac{\alpha\omega BL}{Q}}+\frac{Q}{\alpha\omega BL}\cdot\left( S_{*0}-S_{*} \right)\cdot(1-e^{-\frac{\alpha\omega BL}{Q}})$$
