# Supplementary material for: Assessment of the Multi-Objective Reservoir Operation for Maintaining the Turbidity Maximum Zone in the Yangtze River Estuary
Source: Int J Environ Res Public Health. 2018 Sep 26;15(10):2118. doi: 10.3390/ijerph15102118 (PMC6210024; doi:10.3390/ijerph15102118)
Supplement: Supplementary file 1 [file ijerph-15-02118-s001.zip › File S2.docx]

**File S2. The sediment settling velocity** $\boldsymbol{\omega}_{\boldsymbol{j}}$ **for each size class can be computed individually as follows:**

1) For grain sizes less than 0.15 mm, the sediment settling velocity adopts the Stokes formula:

$\omega_{j}=\frac{g{D_{j}}^{2}}{18\nu}(\frac{\gamma_{s}-\gamma}{\gamma})$ (38)

where $\gamma_{s}$ and $\gamma$ are the bulk densities of sediment and clear water, respectively. $\nu$ represents the kinematic viscosity, which can be obtained by

$\nu=0.01775/(1+0.0337*T+0.000221*T^{2})$ (39)

where $T$ is the water temperature (°C).

2) When grain sizes are 0.15-1.5 mm, $\omega_{j}$ is determined using an empirical formula relevant to the settling transition zone:

$\omega_{j}=6.77\frac{\rho_{s}-\rho}{\rho}D_{j}+\frac{\rho_{s}-\rho}{1.92\rho}(\frac{T}{26}-1)$ (40)

where $\rho_{s}$ and $\rho$ are the densities of sediment and water, respectively.

3) For grain sizes greater than 1.5 mm, $\omega_{j}$ can be obtained from

$\omega_{j}=1.068 \sqrt{\frac{\rho_{s}-\rho}{\rho}gD_{j}}$ (41)
